# A look at post-pandemic conjugality: a clinical case study

**DOI:** 10.1192/j.eurpsy.2023.1651

**Published:** 2023-07-19

**Authors:** C. Pires-Lima, M. H. Figueiredo, A. Salgueiro

**Affiliations:** 1 CINTESIS - Center for Health Technology and Services Research, Porto; 2 Independente, Valença, Portugal

## Abstract

**Introduction:**

The co-construction of conjugality is influenced by the interactions established in the family as a whole. It manifests itself, therefore, as a relational model in the expression of affectivity and the management of conflicts.

**Objectives:**

In this clinical case report, the couple assumes relational difficulties focused on the significant reduction of time in individual leisure activities and, on the other hand, a 24-hour coexistence in the same space, in a period of compulsory confinement due to the COVID-19 pandemic.

**Methods:**

This study is exploratory-descriptive, using the case study as an empirical approach.

**Results:**

The case described, reports to Couples Therapy, with the sessions taking place in 2022, in a total of seven. The couple, N, male, and J, female, have been married since 2020, shortly before the first confinement because of the COVID-19 pandemic. Regarding their marital relationship, they reported that it deteriorated due to the difficulty in expressing an adaptive reaction to the stressor confinement and the opposite position regarding their desire to become parents.

**Conclusions:**

Integrative strategies were developed, with different theoretical and operative references. The couple took control of their relationship due to a greater empathic awareness and the establishment of a healthy and balanced communication system.

**Disclosure of Interest:**

None Declared

